# Periconceptional Heat Stress of Holstein Dams Is Associated with Differences in Daughter Milk Production and Composition during Multiple Lactations

**DOI:** 10.1371/journal.pone.0133574

**Published:** 2015-10-23

**Authors:** Britni M. Brown, Jon W. Stallings, John S. Clay, Michelle L. Rhoads

**Affiliations:** 1 Department of Animal and Poultry Sciences, Virginia Polytechnic Institute and State University, Blacksburg, VA, United States of America; 2 Department of Statistics, North Carolina State University, Raleigh, NC, United States of America; 3 Department of Animal Science, North Carolina State University, Raleigh, NC, United States of America; Van Andel Institute, UNITED STATES

## Abstract

Heat stress at the time of conception affects the subsequent milk production of primiparous Holstein cows; however, it is unknown whether these effects are maintained across multiple lactations. Therefore, the objective of the current study was to examine the relationship between periconceptional heat stress and measurements of milk production and composition in cows retained within a herd for multiple lactations. National Dairy Herd Improvement Association data was obtained from Dairy Records Management Systems. Records included milk production data and milk composition data from over 75,000 and 44,000 Holstein cows, respectively, born between 2000 and 2010 in Florida, Georgia, and Texas. Conception dates were calculated by subtracting 276 d from the recorded birth date. Records for cows conceived within the months of June, July, and August were retained as heat stress conceived (HSC) cows; cows conceived within the months of December, January, and February were retained as thermoneutral conceived (TNC) contemporaries. Adjusted 305-d mature equivalent milk, protein percent and fat percent were evaluated with a mixed model ANOVA using SAS. Milk production was significantly affected by periconceptional heat stress. When a significant difference or tendency for a difference was detected between the HSC and TNC cows, the TNC produced more milk in all but one comparison. The advantage in milk production for the TNC cows over the HSC cows ranged from 82 ± 42 to 399 ± 61 kg per lactation. Alterations in fat and protein percentage were variable and most often detected in first lactations (first > second or third). Overall, the most striking result of this study is the consistency of the relationship between HSC and milk production. The nature of this relationship suggests that heat stress at or around the time of conception impairs cow milk yield throughout her lifetime.

## Introduction

The reduction in milk yield that dairy cattle experience during periods of heat stress is attributed to multiple factors, including reduced feed intake and altered metabolism [1–3]. These consequences of heat stress are particularly detrimental for high producing dairy cows that are already under immense metabolic strain. In the United States alone, approximately $1 billion is lost annually as a result of poor performance of dairy cattle during periods of heat stress [4]. One of the most costly factors contributing to this reduction in performance is the substantial decrease in fertility that occurs during heat stress. Nevertheless, some inseminations conducted during heat stress will result in successful pregnancies from which heifer calves are born. It us unknown, however, whether the heat stress experienced by these females around the time they were conceived confers long-lasting effects that alter subsequent milk production capacity.

Research is just beginning to reveal how the quality and characteristics of the periconceptional environment affect long-term productivity of livestock species. Emerging evidence in other species has demonstrated the importance of the periconceptional environment for health and performance during adulthood [5, 6]. In sheep, nutritional stress (under- or over-nutrition) around the time of conception has been linked with postnatal alterations in physiology and behavior [7, 8]; some of which have been shown to persist into adulthood. If periconceptional stress of dairy cattle causes comparable alterations, lifetime productivity and profitability would ultimately be disrupted.

Periconceptional heat stress and the resulting physiological consequences are likely capable of modifying life-long productive capacity of dairy cattle. Unfortunately, the magnitude of these effects is poorly understood at this time; especially those effects that persist for multiple lactations. Therefore, the objective of the present study was to evaluate the effects of periconceptional heat stress on subsequent milk production and composition from cows that were retained within the herd for three or more lactations. The population of cattle in these analyses provides unique insight into the true impact of periconceptional heat stress because they are cattle that were selected for retention within the herd, unlike many of their contemporaries that would have been culled after their first lactation. Consequently, these individuals had multiple opportunities (lactations) to contribute to or detract from herd profitability. For cows that are retained within a herd for multiple lactations, any factor that results in a repetitive reduction in milk yield causes economic loss for the producer. If detectable reductions in milk yield over multiple lactations exist, the implications for productivity should be considered as producers make decisions about which females will be retained as replacement animals.

## Materials and Methods

### Measures of Milk Production and Milk Composition

The milk production variable that was selected for analysis in this study was the 305-day adjusted mature-equivalent milk. This variable was chosen, because amongst other factors, the calculation includes correction factors for the age of the animal at the time of calving. Fat and protein production were analyzed and are presented as a percentage of total milk production. Each was calculated by dividing 305-day adjusted mature-equivalent fat (or protein) by 305-day adjusted mature-equivalent milk, which was then multiplied by 100.

### Inclusion Criteria

Dairy Herd Improvement Association data was received from Dairy Records Management Systems (Raleigh, NC) for three states known to have hot and humid summer conditions: Georgia, Florida, and Texas. In order to be included in the analyses for this study, Holstein cows were required to have their first, second, and third lactation within one herd; in all cases only the first three lactations were evaluated. The milk production analysis further required Holstein cows to have a mature-equivalent milk yield between 2268 and 20412 kg for each lactation period, while the analysis of fat and protein composition required percentages between 2 and 5% and 2 and 3.75%, respectively. These restrictions were necessary to remove outliers and ultimately removed no more than 1% of the total number of records available.

The date that each individual cow was conceived was calculated using their birth date and an average gestation length for Holsteins of 276 d [9]. Whether or not an individual was likely to have experienced heat stress at the time of their conception was determined by the month in which conception took place. Cows that were conceived during December, January, and February were considered thermoneutral conceived (TNC), while cows conceived during June, July, and August were considered heat stress conceived (HSC). Cows conceived during the fall (September, October and November) and spring (March, April, and May) were excluded from the analyses because weather conditions in these seasons are highly variable. Finally, only herds including at least 10 cows were analyzed. For each state, the total numbers of cows included in the analyses are listed in Table 1. The number of cows included in the milk production and composition analyses differed from each other because composition records were not available for every cow with milk production data.

**Table 1:** Number of cows from Georgia, Florida and Texas that were included in the analyses of milk production and composition from thermoneutral-conceived (TNC) and heat stress-conceived (HSC) Holstein cattle for three lactations.

	Georgia		Florida		Texas		Total	
	Milk Production	Milk Composition (Fat/Protein)	Milk Production	Milk Composition(Fat/Protein)	Milk Production	Milk Composition (Fat/Protein)	Milk Production	Milk Composition (Fat/Protein)
TNC	12,099	6,844/6,907	15,027	6,054/6,264	19,498	15,022/15,028	46,624	27,920/28,199
HSC	7,020	3,144/3,177	8,071	3,271/3,374	13,750	9,941/9,945	28,841	16,356/16,496
Total No. of Cows	19,119	9,988/10,084	23,098	9,325/9,638	33,248	24,963/24,973	75,465	44,276/44,695

Exposure to thermoneutral and heat stress conditions during the winter and summer months was verified by calculating the temperature-humidity index (THI) for each season within each state. Climatic data from 1999 through 2011, including hourly temperature, humidity, and dew-point observations, were retrieved from the National Climatic Data Center (http://www.ncdc.noaa.gov/). Temperature-humidity index values were calculated using an equation derived for use with dairy cattle [10].

$$THI=T_{\text{dry bulb}}+\left(\left(0.36*T_{\text{dew point}}\right)+41.2\right)$$

A THI value was calculated for each hourly observation, which was then used to determine the mean THI for each season.

In order to appropriately evaluate milk production and composition, we not only considered the season in which the cow herself had been conceived, but also the season in which she calved. Season of calving (SOC) was determined using the month of each calving date. The seasons were designated as winter: December, January and February; spring: March, April, May; summer: June, July, August; and fall: September, October, November.

### Statistical Analyses

All analyses were performed using PROC MIXED in SAS (SAS Institute Inc., Cary, NC). Each state was analyzed individually. The analysis focused on estimating the effects of heat stress, lactation number, season of calving (SOC), and their interactions on milk production or composition, as appropriate. An effect was included to account for the variation across herds in the state. As the herds may be considered a random sample of all possible herds in the state, these effects were considered to be random, which allowed generalization of inferences to the entire state. We further included each cow as a random effect in the analysis, nested within herd. Due to the large number of random effects, a RANDOM statement was used in PROC MIXED to incorporate the herd random effects while a REPEATED statement with a complete-symmetry covariance structure was used to incorporate the individual cow random effects. Pairwise contrasts comparing HSC and TNC at different lactation numbers and SOC levels were then performed to investigate the interactions. Due to the large sample size, results were considered significant with a P-value ≤0.01, and a tendency for significance was declared at P≤0.05.

## Results

### Temperature-Humidity Index

**Fig 1:**
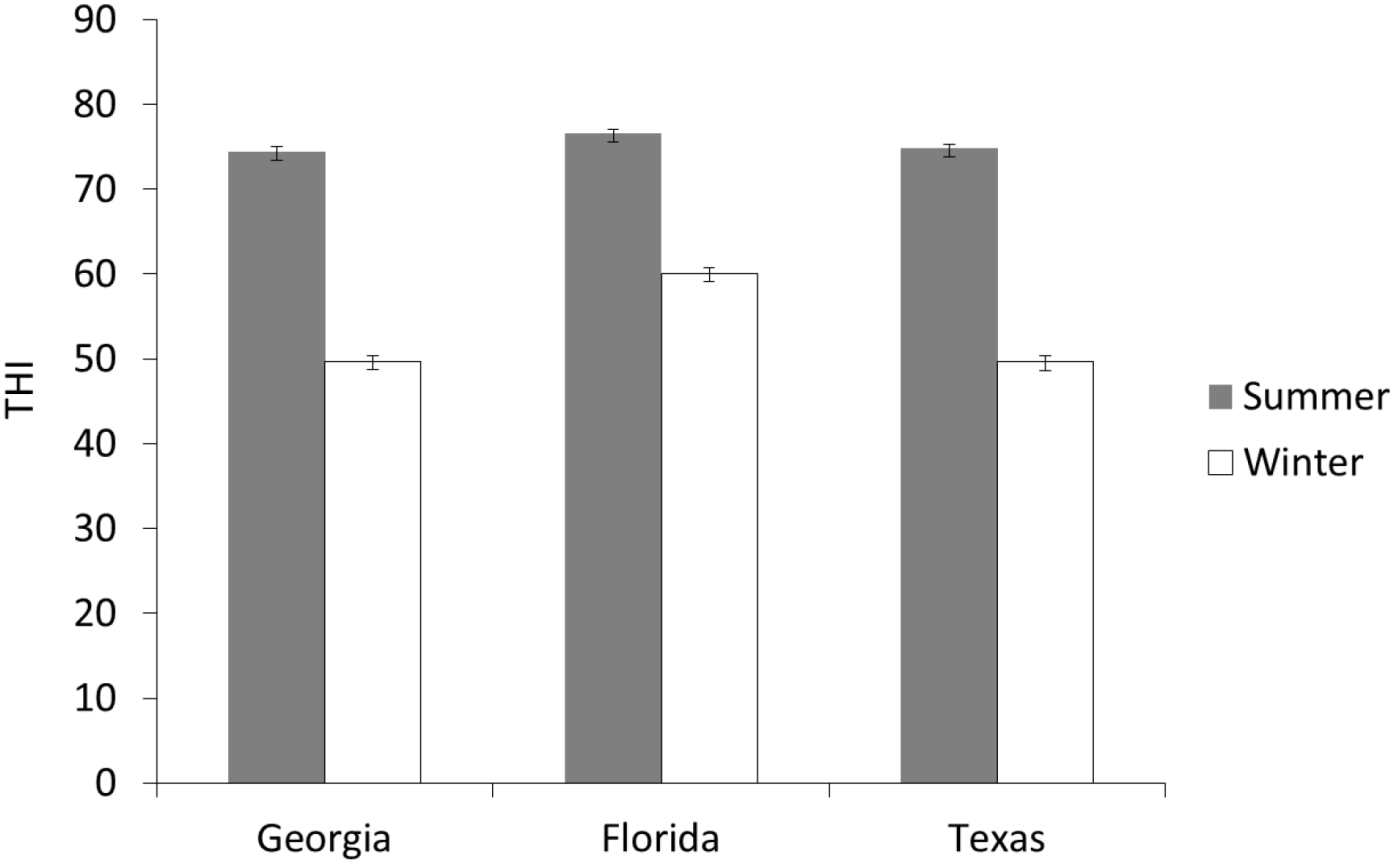
The mean temperature-humidity index (THI) during the summer and winter seasons in Georgia, Florida and Texas. The threshold for heat stress in dairy cattle is a THI value of 68. During summer months, the average THI exceeded the threshold for heat stress in dairy cattle. In winter months, the average THI did not exceed the threshold.

Traditionally, a THI value of 72 has been used as a threshold to predict whether or not dairy cattle are experiencing heat stress; meaning that at a THI of 72 or above, producers should expect a decrease in milk yield. However, this threshold was based upon a retrospective analysis of studies conducted in the 1950’s and 1960’s. A recent re-evaluation of the THI indicates that high-producing dairy cattle are much more sensitive to environmental conditions than previously thought. Milk production actually begins to decline when the minimum THI does not fall below 65, or the average THI is 68 or greater [11]. The average seasonal THI values were calculated for each state used in this study, and are shown in Fig 1. Regardless of which threshold is used as a designation of heat stress (THI of 72 or 68), it is clear that, overall, summer conditions exceeded the threshold while winter conditions did not.

### Milk Production

**Fig 2:**
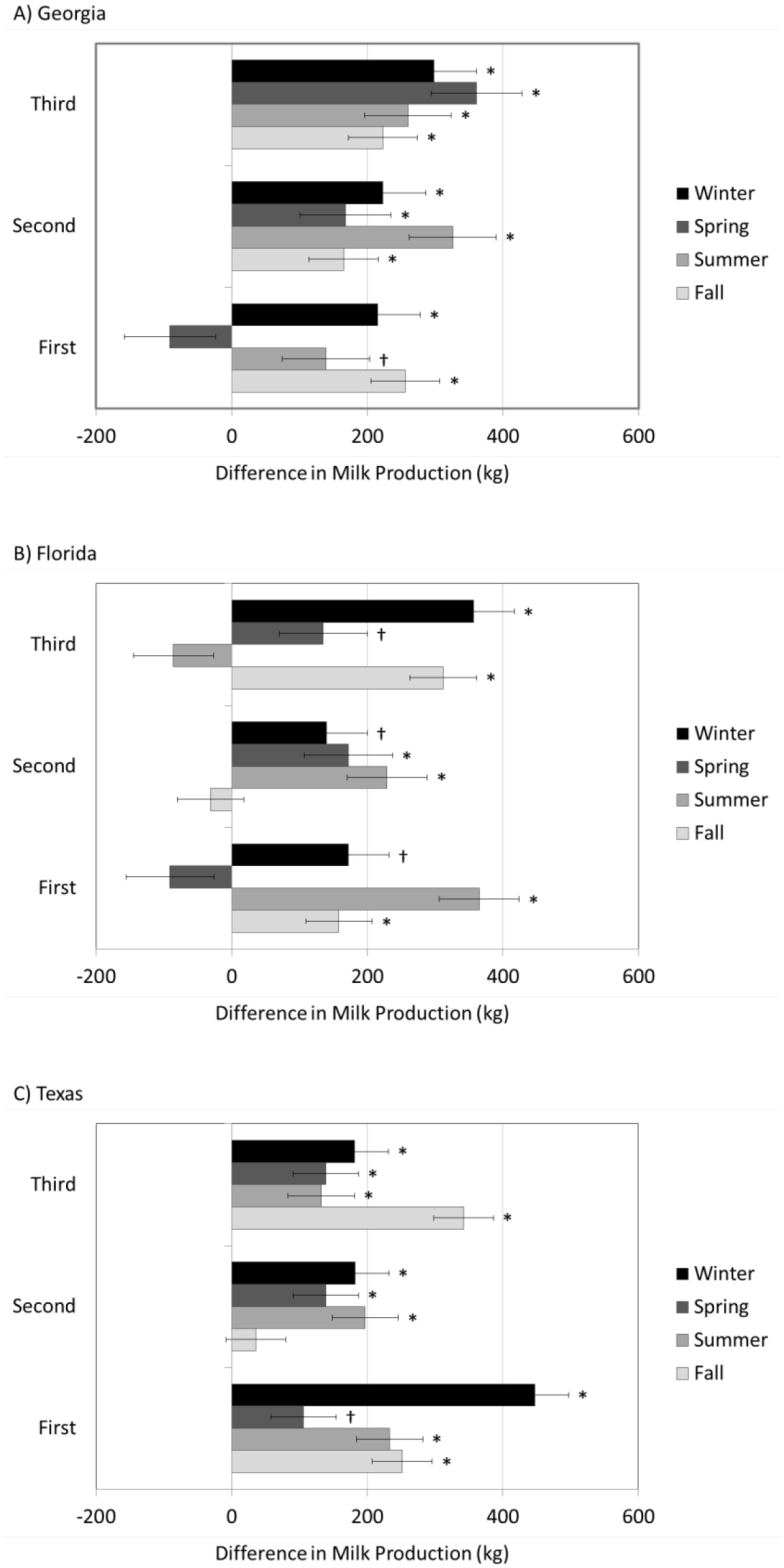
Differences in mature-equivalent milk yield (kg) between thermoneutral conceived (TNC) and heat stress conceived (HSC) cows in Georgia (A), Florida (B) and Texas (C) during their first, second and third lactations. In instances where TNC cows produced more milk than their HSC counterparts, those values are positive. In instances where HSC cows produced more milk than their TNC counterparts, those values are negative. Bars with * denotes a significant difference (P<0.01) while † denotes a tendency for a difference (P<0.05).

For all three states, milk production was associated with the three-way interaction between the periconceptional environment (TNC or HSC), lactation number and SOC (P<0.01). The results of the contrasts within lactation number and SOC were remarkably consistent (most indicating TNC cows produced more milk), and the majority of the contrasts resulted in the detection of significant differences in milk production between the TNC and HSC cows (Fig 2). Of the 36 comparisons, 26 were significantly different, and an additional 5 comparisons tended to differ. The TNC cows produced more milk than the HSC cows in every instance where a significant difference was detected, and for most comparisons (4 out of 5) where a tendency for a difference was detected (regardless of location, lactation number or SOC). The advantage in milk production for the TNC cows over the HSC cows ranged from 82 ± 42 to 399 ± 61 kg per lactation.

### Milk Fat Percentage

For Georgia and Texas, there was an effect of lactation number on the percentage of fat produced (P<0.01; data not shown). In Texas, there was a significant interaction between periconceptional environment and season of calving (P<0.01). Contrasts revealed that HSC cows that calved during the fall and winter tended to have a higher fat percentage than TSC cows by 0.03 ± 0.01 and 0.05 ± 0.01, respectively (P<0.01). There were no three-way interactions of periconceptional environment (TNC or HSC), lactation number and SOC in Georgia or Texas, so this trend was consistent across lactations.

Interestingly, for Florida there was a three-way interaction between periconceptional environment (TNC or HSC), lactation number and SOC (P<0.01). Contrasts revealed six instances in which the percentage of fat produced differed or tended to differ (Table 2). Most of those instances occurred during the first lactation (four out of six contrasts) and in most cases, it was the TNC cows that produced milk with a higher fat content (four out of six contrasts).

**Table 2:** Differences in milk fat percentage points between thermoneutral conceived (TNC) and heat stress conceived (HSC) cows in Florida.

Lactation	SOC^1^	% Fat Difference^2^	S.E.	P-Value
1	Spring	0.06	0.02	<0.01
	Summer	-0.04	0.02	0.04
	Fall	-0.10	0.02	<0.01
	Winter	0.09	0.03	<0.01
2	Spring	0.07	0.02	<0.01
	Summer	0.02	0.02	0.37
	Fall	<0.01	0.02	0.85
	Winter	0.02	0.02	0.25
3	Spring	0.04	0.02	0.10
	Summer	0.05	0.02	0.01
	Fall	-0.03	0.02	0.07
	Winter	<0.01	0.02	0.92

^1^Season of Calving (SOC)

^2^Positive values indicate the superior performance of TNC cows, while negative values indicate the superior performance of HSC cows.

### Milk Protein Percentage

For all three states, protein percentages exhibited a significant three-way interaction of periconceptional environment (TNC or HSC), lactation number and SOC (P<0.01). Similar to the results of the analyses of milk fat percentage, significant differences between TNC and HSC cows in Georgia and Florida were detected primarily during the first lactation. Differences were observed in every lactation period for Texas. Table 3 contains differences in the first lactation period for Georgia and Florida and all three lactation periods for Texas. Unlike the results of the other analyses however, for those contrasts that differed or tended to differ, it was more often the HSC cows that produced a greater percentage of milk protein rather than the TNC cows (twelve of the fifteen contrasts that differed or tended to differ).

**Table 3:** Differences in milk protein percentage points between thermoneutral conceived (TNC) and heat stress conceived (HSC) cows in Georgia and Florida.

State	Lactation	SOC^1^	Percent Protein^2^ Produced^1^ (%)	S.E.	P-Value
Georgia	1	Spring	0.03	0.007	<0.01
		Summer	0.01	0.008	0.18
		Fall	-0.03	0.007	<0.01
		Winter	-0.02	0.009	0.07
Florida	1	Spring	0.04	0.009	<0.01
		Summer	-0.03	0.009	<0.01
		Fall	-0.03	0.009	<0.01
		Winter	-0.01	0.01	0.11
Texas	1	Spring	0.02	0.005	<0.01
		Summer	<0.01	0.007	0.66
		Fall	-0.02	0.006	<0.01
		Winter	-0.02	0.008	0.04
	2	Spring	-0.03	0.006	<0.01
		Summer	-0.01	0.005	0.29
		Fall	-0.01	0.004	0.04
		Winter	-0.03	0.006	<0.01
	3	Spring	-0.02	0.006	<0.01
		Summer	-0.02	0.005	<0.01
		Fall	-0.02	0.004	<0.01
		Winter	-0.02	0.005	<0.01

^1^Season of Calving (SOC)

^2^Positive values indicate the superior performance of TNC cows, while negative values indicate the superior performance of HSC cows.

## Discussion

As research broadens our understanding of the physiological response to heat stress, it has become apparent that the interactions between heat stress, milk production and milk composition are more complex than once thought. During this periconceptional period (and during other life stages), the cellular and molecular changes initiated as a result of exposure to stressful conditions are adaptive mechanisms that allow cells to continue to survive. These alterations are of particular interest for dairy cattle because, theoretically, they can be retained in the genome [12] and cause life-long consequences for productivity. Alternatively (or simultaneously) the cellular and molecular changes that occur in response to heat stress may well affect the anatomy and physiology of the individual. For example, the cellular and molecular response to heat stress could alter the development of organs, endocrine systems and patterns of vasculogenesis and/or angiogenesis. Although the potential mechanisms involved in the putative relationship between periconceptional heat stress and productivity are intriguing, it was necessary to first prove that a long-term association exists. To our knowledge, this is the first study to demonstrate differences in milk production and composition emanating from heat stress that was experienced at the time of conception.

The results of this study reveal a remarkably consistent association between periconceptional heat stress and milk production across states, lactation numbers and SOC. In almost all instances, there was a difference or tendency for a difference in milk production based upon whether or not the cow had been conceived in the winter (TNC) or summer (HSC) months. More importantly, in every instance when a difference was detected and in most instances where a tendency for a difference was detected, it was the TNC cows that produced more milk than their HSC counterparts. In some cases the deficit in milk produced by the HSC cows was substantial, and in other cases the difference was relatively modest. Regardless of the magnitude, however, it is important to recognize that these associations exist and persist across multiple lactations in a population of cows that have been subjected to substantial selection pressure.

While the current study was focused on the effects of heat stress during the periconceptional period, previous research has shown that exposure to heat stress during late gestation causes a multitude of adverse consequences for both the dairy cow and her offspring. Of those consequences, the ones most pertinent to the current study would be the resultant effects on the offspring (because in each case, those would be consequences of prenatal heat stress). Indeed, calves exposed to late gestational heat stress exhibit altered characteristics of metabolism and immune function. Those effects are comprehensively reviewed in Tao and Dahl [13]. Unfortunately, however, milk production capacity of females exposed to late gestational heat stress has not been reported. Few, if any dams included in the current study were prenatally exposed to late gestational heat stress because of the inclusion criteria for the study: conceived during either winter or summer, so born in fall or spring. For the dry cow, however, one of the most costly results of late gestational heat stress is an observed reduction in milk yield during the subsequent lactation [14, 15]. This makes one wonder whether a female that was prenatally exposed to heat stress during both the periconceptional period and late gestation would produce even less milk than counterparts that had only been exposed to a single insult (periconceptional or late gestation).

From state to state, the nature of the relationship between periconceptional heat stress and milk production was similar. For Georgia and Texas, there were very few instances where the TNC cows did not produce more milk than the HSC cows (one or two instances each where there was neither significance nor a tendency; Fig 2). In Florida, however, there was slightly more variation with two occasions in which milk production between TNC and HSC cows did not differ and three other instances where there was only a tendency for TNC cows to produce more milk. Of the comparisons that differed or tended to differ, Florida is also the only of the three states where HSC cows produced more than TNC cows (one instance). This is interesting because of all three states that were included in these analyses, Florida has the warmest winter temperatures. Therefore, even though TNC cows were conceived during the winter months of December, January and February, TNC cows from Florida were more likely to have been exposed to acute mild or moderate heat stress during the periconceptional period. This factor may have contributed to the slightly greater variability in milk production response.

Although the trends for effects of periconceptional heat stress on milk production were relatively similar across states, lactations and SOC, effects on milk composition were much more variable. Fat and protein percentages were found to differ between TNC and HSC cows more frequently in first lactation animals than those in their second or third lactation. The apparent disappearance of effects is likely a consequence of parity-induced changes in milk, protein and fat production. With each successive lactation, dairy cattle produce more milk, and therefore as a percentage, will produce less protein and perhaps less fat. Consequently, as dairy cattle enter their third lactation, lower mean percentages overall make it more difficult to detect statistical differences in milk composition than it would be for their first lactation.

Heat stress of mature dairy cattle has been shown to alter milk fat and protein proportions in previous studies, but this is generally attributed to seasonal alterations in forage availability and depressed nutrient acquisition resulting from reduced feed intake [16, 17]. Variation in forage quality would not have affected the results reported here since TNC and HSC cattle would have been consuming the same diets within a season. Unfortunately, there is no way to know whether TNC cows and HSC cows consumed comparable quantities of feed and/or nutrients within seasons. In utero heat stress is known to affect animal physiology in sometimes unexpected ways. For example, pigs whose dams were exposed to heat stress during gestation have a lower skin to BW ratio. This reduction is likely due to decreased skin thickness and may improve their ability to dissipate heat [18]. If a similar adaptation occurs in HSC cattle, they may be more thermotolerant and therefore better able to maintain feed intake during periods of heat stress.

Overall, the most striking result of this study was the consistent association between periconceptional heat stress and milk production. Although the physiological mechanisms involved in this association remain unknown, these results provide the basis for future targeted studies. Regardless of the mechanisms involved, lower production potential is an economic loss that could be avoided by circumventing periconceptional heat stress.
