# Correction: Periconceptional Heat Stress of Holstein Dams Is Associated with Differences in Daughter Milk Production and Composition during Multiple Lactations

**DOI:** 10.1371/journal.pone.0150049

**Published:** 2016-02-22

**Authors:** Britni M. Brown, Jon W. Stallings, John S. Clay, Michelle L. Rhoads

Fig 2 appears incorrectly in the published article. Please see the correct Fig 2 and its legend below.

**Fig 2:**
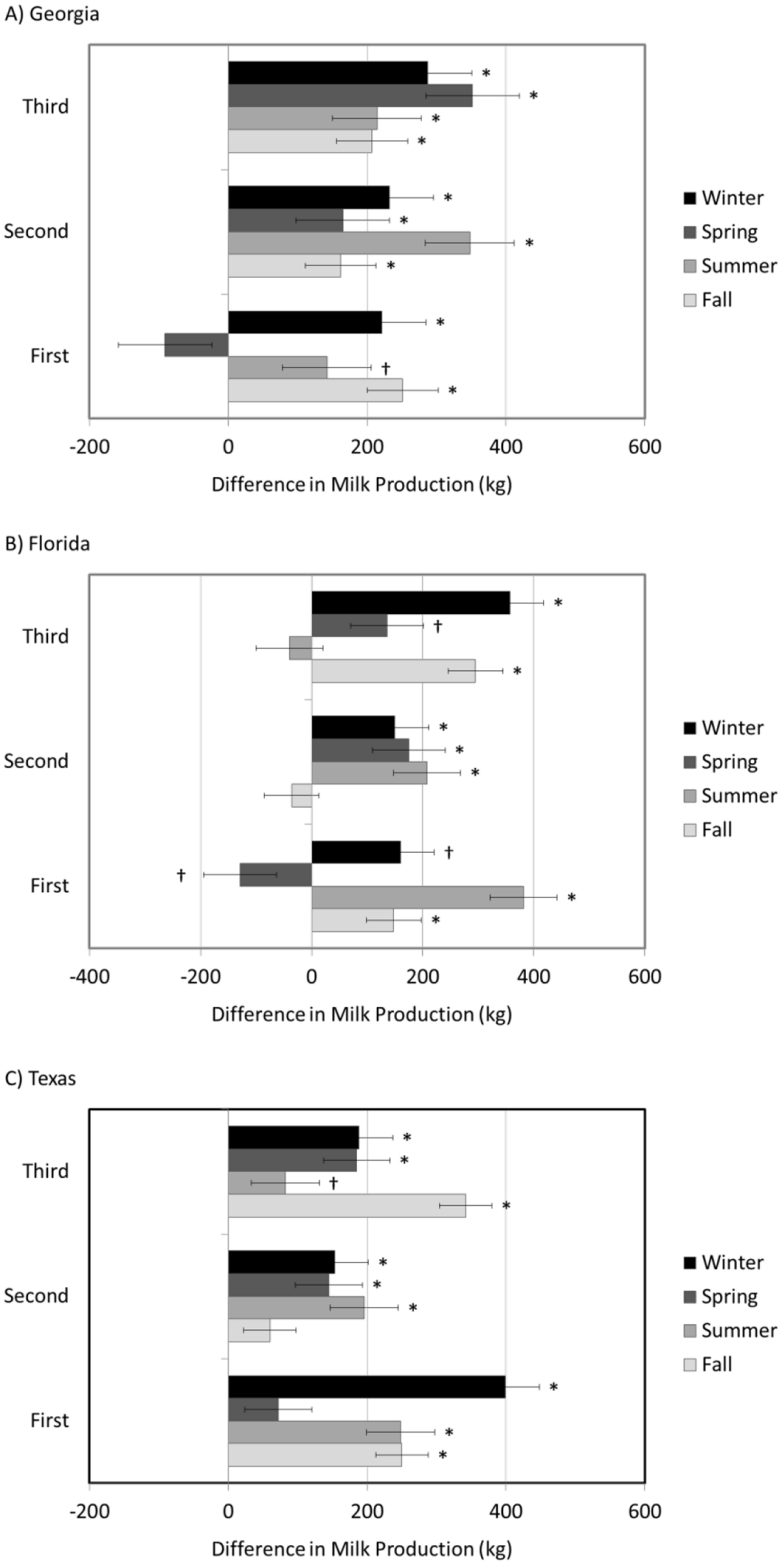
Differences in mature-equivalent milk yield (kg) between thermoneutral conceived (TNC) and heat stress conceived (HSC) cows in Georgia (A), Florida (B) and Texas (C) during their first, second and third lactations. In instances where TNC cows produced more milk than their HSC counterparts, those values are positive. In instances where HSC cows produced more milk than their TNC counterparts, those values are negative. Bars with * denotes a significant difference (P<0.01) while † denotes a tendency for a difference (P<0.05).
